# Editorial: Progress in neuroacanthocytosis syndromes and related diseases including other bulk lipid transfer disorders

**DOI:** 10.3389/fnins.2025.1635008

**Published:** 2025-06-30

**Authors:** Adrian Danek, Andreas Hermann, Lars Kaestner, Mercè Masana, Kevin Peikert, Manuel J. Rodriguez, Ruth H. Walker

**Affiliations:** ^1^Neurologische Klinik und Poliklinik, LMU Klinikum, LMU Munich, Munich, Germany; ^2^Translational Neurodegeneration Section “Albrecht Kossel”, Department of Neurology, University Medical Center Rostock, University of Rostock, Rostock, Germany; ^3^Center for Transdisciplinary Neurosciences Rostock (CTNR), University Medical Center Rostock, Rostock, Germany; ^4^United Neuroscience Campus Lund-Rostock (UNC), Rostock, Germany; ^5^Theoretical Medicine and Biosciences, Medical Faculty, Saarland University, Homburg, Germany; ^6^Dynamics of Fluids, Experimental Physics, Faculty of Natural Science and Technology, Saarland University, Saarbrücken, Germany; ^7^Department of Biomedical Sciences, School of Medicine and Health Sciences, Institute of Neurosciences, Universitat de Barcelona, Barcelona, Spain; ^8^August Pi i Sunyer Biomedical Research Institute (IDIBAPS), Barcelona, Spain; ^9^Networked Biomedical Research Centre for Neurodegenerative Disorders (CIBERNED), Madrid, Spain; ^10^Department of Neurology, James J. Peters Veterans Affairs Medical Center, Bronx, NY, United States; ^11^Department of Neurology, Icahn School of Medicine at Mount Sinai, New York City, NY, United States

**Keywords:** BLTP, VPS13, XK, neurodegeneration, neurodevelopment, neuroacanthocytosis

The rare conditions XK disease (McLeod syndrome) and VPS13A disease (chorea-acanthocytosis) have historically been termed “neuroacanthocytosis” due to the association of neurodegeneration, particularly of the basal ganglia, with spiky deformed red blood cells (acanthocytes). Even more obsolete is the 1960s' designation “Levine-Critchley syndrome”: genetic analyses of the reported families have determined that Levine's patients were affected by mutations of *XK* and those of Critchley by *VPS13A* variants. Two multi-author books summarize early developments (Danek, 2004; Walker et al., 2008).

Clinical updates regarding VPS13A disease and XK disease are regularly provided in open access resources (Peikert et al., 2023; Jung et al., 2021). Typical presentations are in young adults of any sex, often siblings (due to autosomal-recessive *VPS13A* mutations), or in middle-aged men, often maternal uncles and nephews (due to X-linked *XK* mutations). In addition to disorders of movement, cognition, and behavior (“huntingtonism”), there may be seizures, neuropathy, and myopathy. Impairment of activities of daily life and a reduced life span are typical, however clinical features can vary considerably. The presence of acanthocytosis is unreliable as a biomarker and, unfortunately, erythrocyte sedimentation rate prolongation as well as creatine kinase elevation, typical in both conditions, are non-specific and easily overlooked. The two conditions' close resemblance is likely explained by the recent observations that the XK and VPS13A proteins interact to enable bulk lipid transport at contact sites between various cell organelles as well as the cell membrane.

Testing at the DNA or protein level (genes: *VPS13A* and *XK*; proteins: VPS13A/chorein and Kell complex red cell antigen Kx) clearly distinguishes the two conditions, however, diagnostic delay continues to be a significant issue due to a variety of factors, including access to molecular testing. It is critical to use the genetic nomenclature rather than the historical term “neuroacanthocytosis” to ensure that patients receiving these diagnoses have been confirmed to carry causative variants of the responsible genes. As we enter the era of molecular treatments, this research topic becomes even more critical.

The VPS13 family comprises additional proteins involved in neurodegeneration (VPS13C, VPS13D) and neurodevelopment (VPS13B, associated with Cohen syndrome) and belongs to the newly recognized superfamily of bridge-like lipid transfer proteins (BLTPs; within this nomenclature, the VPS13 proteins are known as BLTP5A-BLTP5D). This family also includes “Tweek” (BLTP1, involved in a neurodevelopmental disorder) and “Hobbit” (BLTP2). Research in this field has rapidly taken off and flourishes (Levine and Conibear, 2024; Hanna et al., 2023; Reinisch et al., 2025).

Below we summarize the contributions to the Research Topic “Progress in Neuroacanthocytosis Syndromes and Related Diseases Including other Bulk Lipid Transfer Disorders” that grew out of the 11^th^ International Neuroacanthocytosis Symposium (Kaestner, 2023). Further contributions resulted from the “VPS13 forum,” a teleconferencing discussion format that developed with the COVID-19 pandemic (Peikert and Danek, 2023).

Swan exhaustively reviews the BLTP family as well as its members' respective proteins and asks essential questions about their transport function; whether it is just unidirectional or works both ways, how selective for lipid type it is, what drives it, and how it may be regulated. Neiman elaborates this perspective: molecular dysfunction might be treated with transport-enhancing molecules or with bypassing agents. Naturally existing bypasses or duplicate pathways in some of the congenital BLTP diseases offer excellent explanations for delays of their onset into early (VPS13A) and even late (XK) adulthood, while non-viability with complete *VPS13D* knockdown (Seong et al., 2018) marks certain lipid transport pathways as essential for survival: their loss cannot be compensated for.

Osmotic gradient ektacytometry of erythrocytes for diagnostic screening was comprehensively explored by Hernández et al.. Along with microfluidic assays the approach is promising, however sensitivity, specificity, and availability of these novel screening test candidates need further elaboration. Ektacytometry in a complex single case of VPS13A disease who also harbored a *CANVAS* mutation confirmed its usefulness as well as in another VPS13A case and a patient with XK disease, but, as reported by Paucar et al., was normal in acanthocytosis associated with liver disease. Based on their single case observation, Xu et al. review the status of treating VPS13A disease symptomatically with deep brain stimulation.

The presentations of XK and VPS13A diseases with a clinical phenotype similar to Huntington's disease (HD) invite a comparison between these disorders, which share the feature of caudate nucleus vulnerability. García-García et al. undertook one such study and in both, HD transgenic mouse and HD human brain, found VPS13A distribution and expression essentially unaltered.

Turning to XK disease presentation, Walker et al. report four new cases with proven mutations and typical Kell blood group findings, and provide follow-up on members from two known families. Dambietz et al. report on the protracted diagnostic process in one patient with cerebrospinal fluid T-cell abnormalities. If confirmed in absence of COVID-19, this novel finding suggests as yet unexplored functions of XK (see also Tanti et al., 2024).

Complementing the neurological, psychiatric, cardiologic, and hematologic perspectives of BLTP diseases, Schottmann et al. focus on the pediatric and ophthalmological condition of Cohen syndrome and on intricacies of its genetic testing and prediction. In their family with two clinically affected members four rare *VPS13B* variants were detected. Of one in particular, the disease-causing property was unclear until it underwent functional testing. Vacca et al. review the molecular mechanisms possibly underlying VPS13B disease, focusing on studies in mice and the naturally occurring VPS13B disease model of Border Collies (“trapped neutrophil syndrome”).

The articles collected here represent a number of recent advances in this rapidly-evolving field. The ultimate goal is to develop and validate therapies for these devastating neurological and multi-organ disorders, and additionally to add to understanding of cellular processes which may benefit other fields.

## References

[B1] Danek A. (2004). Neuroacanthocytosis Syndromes. Dordrecht, The Netherlands: Springer. 10.1007/1-4020-2898-9

[B2] Hanna M. Guillén-Samander A. De Camilli P. (2023). RBG motif bridge-like lipid transport proteins: structure, functions, and open questions. Annu. Rev. Cell Dev. Biol. 39, 409–434. 10.1146/annurev-cellbio-120420-014634 37406299

[B3] Jung H. H. Danek A. Walker R. H. Frey B. M. Peikert K. (2021). “McLeod neuroacanthocytosis syndrome,” in GeneReviews^®^ (Seattle, WA: University of Washington, Seattle). Available online at: http://www.ncbi.nlm.nih.gov/books/NBK1354/20301528

[B4] Kaestner L. (2023). Proceedings of the eleventh international meeting on neuroacanthocytosis syndromes. Tremor Other Hyperkinet Mov 13:41. 10.5334/tohm.826 37928888 PMC10624129

[B5] Levine T. P. Conibear L. (2024). Editorial on special collection in contact: VPS13 and bridge-like lipid transfer proteins: a new mode of intracellular continuity. CONTACT 7:25152564241238538. 10.1177/25152564241238538 38496780 PMC10943745

[B6] Peikert K. Danek A. (2023). VPS13 forum proceedings: XK, XK-related and VPS13 proteins in membrane lipid dynamics. Contact 6:25152564231156994. 10.1177/25152564231156994 37366410 PMC10243564

[B7] Peikert K. Dobson-Stone C. Rampoldi L. Miltenberger-Miltenyi G. Neiman A. De Camilli P.. (2023). “VPS13A Disease,” in GeneReviews^®^, (Seattle, WA: University of Washington, Seattle). Available online at: http://www.ncbi.nlm.nih.gov/books/NBK1387/20301561

[B8] Reinisch K. M. De Camilli P. Melia T. J. (2025). Lipid dynamics at membrane contact sites. Annu. Rev. Biochem. 10.1146/annurev-biochem-083024-122821. [Epub ahead of print]. 40067957

[B9] Seong E. Insolera R. Dulovic M. Kamsteeg E.-J. Trinh J. Brüggemann N.. (2018). Mutations in *VPS13D* lead to a new recessive ataxia with spasticity and mitochondrial defects. Ann. Neurol. 83, 1075–1088. 10.1002/ana.25220 29604224 PMC6105379

[B10] Tanti G. K. Brill M. L. Kalluri S. Srivastava R. Lepennetier G. Öllinger R.. (2024). “Xk is a novel oligodendrocyte protein with a potential role in de/remyelination,” in Program No. PSTR325.01. 2024 Neuroscience Meeting Planner. Chicago, IL: Society for Neuroscience. Available online at: https://www.abstractsonline.com/pp8/#!/20433/presentation/19115

[B11] Walker R. H. Saiki S. Danek A. (2008). Neuroacanthocytosis Syndromes II. Berlin Heidelberg, Germany: Springer. 10.1007/978-3-540-71693-8

